# *Artemisia Iwayomogi* Extract Attenuates High-Fat Diet-Induced Hypertriglyceridemia in Mice: Potential Involvement of the Adiponectin-AMPK Pathway and Very Low Density Lipoprotein Assembly in the Liver

**DOI:** 10.3390/ijms18081762

**Published:** 2017-08-12

**Authors:** Jinhui Lee, Vikram P. Narayan, Eun Young Hong, Wan Kyunn Whang, Taesun Park

**Affiliations:** 1 Department of Food and Nutrition, Brain Korea 21 PLUS Project, Yonsei University, 50 Yonsei-ro, Seodaemun-gu, Seoul 03722, Korea; Jinhui9101@naver.com (J.L.); narayan.v.2012@gmail.com (V.P.N.); 2 Pharmaceutical Botany Laboratory, College of Pharmacy, Chung-Ang University, Heukseok-dong, Dongjak-gu, Seoul 06974, Korea; hrosal2001@naver.com (E.Y.H.); whang-wk@cau.ac.kr (W.K.W.)

**Keywords:** *Artemisia iwayomogi*, hypertriglyceridemia, adiponectin, lipogenesis, fatty acid oxidation, very low density lipoprotein synthesis

## Abstract

This study aimed to examine the protective effect of *Artemisia iwayomogi* extract (AI) against hypertriglyceridemia induced by a high-fat diet (HFD) in mice and to uncover the underlying molecular mechanisms. C57BL/6N mice were fed chow, HFD, HFD + 0.1% AI, HFD + 0.25% AI, or HFD + 0.5% AI for 10 weeks. The addition of 0.25% and 0.5% AI resulted in dose-dependent improvements in the major parameters of hypertriglyceridemia, including plasma triglyceride, free fatty acids, apolipoprotein B, and lipoprotein lipase, with parallel reductions in body weight gain, hepatic lipid accumulation, and insulin resistance. These beneficial effects were accompanied by the activation of adiponectin-adenosine monophosphate-activated protein kinase (AMPK) mediated signaling cascades in the liver, which downregulated molecules involved in lipogenesis and concurrently upregulated molecules related to fatty acid oxidation. The downregulation of molecules involved in very low density lipoprotein assembly, which was associated with improved hepatic insulin signaling, also appeared to contribute to the AI-induced attenuation of hypertriglyceridemia.

## 1. Introduction

Hypertriglyceridemia refers to a fasting plasma triglyceride (TG) concentration that typically increased above the 95th percentile for age and sex in the population, along with the presence of additional quantitative or qualitative lipoprotein abnormalities [1,2]. The elevated plasma TG concentrations are predominantly seen in disorders characterized by insulin resistance, central obesity, and metabolic syndrome [3] and are considered as an independent risk factor for atherosclerotic cardiovascular disease [4]. Plasma TGs can be obtained from exogenous sources such as dietary fat and are carried in chylomicrons or they can be obtained from endogenous sources such as the liver and are carried in very low density lipoprotein (VLDL) particles. In capillaries within adipose and muscle tissues, these lipoproteins and chylomicrons are hydrolyzed by lipoprotein lipase (LPL) into free fatty acids (FFAs). Experimental animals have been subjected to a high-fat diet (HFD) to induce hypertriglyceridemia with concomitant visceral adiposity, hepatic steatosis, and insulin resistance. The abnormal concentration of TG-rich lipoproteins in the fasting plasma of HFD-fed rodents resulted from upregulated lipogenic and secretory pathways and/or decreased peripheral catabolism, which occurred mainly through reduced LPL activity. Additionally, the insulin resistance observed in HFD-fed animals drove the increased flux of FFAs into hepatocytes and stimulated VLDL synthesis and secretion by the liver [5], which contributed to hypertriglyceridemia.

The general treatment regimen for hypertriglyceridemia includes lifestyle changes and dietary modifications. When these have not been successful, drug treatments, including fibrates, omega-3 fatty acids, and nicotinic acid, alone or in combination with statins, are also used [6]. The potential of natural products to treat hypertriglyceridemia is still largely unexplored and might offer alternative remedies. *Artemisia iwayomogi* is a perennial aromatic plant with yellow flowers, distributed throughout Korea. The aerial parts of *A. iwayomogi* have been used in oriental medicine for centuries to cure various infectious diseases such as carbuncles, cholecystitis, hepatitis, and jaundice [7,8]. A hepatoprotective effect of *A. iwayomogi* extract was reported in rodent models with different types of liver diseases, such as alcoholic fatty liver [9], carbon tetrachloride-induced chronic hepatic fibrosis [10,11], and cholestatic liver fibrosis [12]. We previously reported that *A. iwayomogi* extract (0.5% *w*/*w* diet) reduced visceral fat accumulation and obesity-related biomarkers in mice fed a HFD by modulating the expression of genes associated with adipogenesis and inflammation in adipose tissues [13]. Concurrently in this extract-fed mice, we also observed a decrease in plasma TG levels, however the underlying mechanism responsible was not elucidated. A more recent study reported that a mixture (0.1% *w*/*w* diet) of *A. iwayomogi* and *Curcuma longa* extracts provoked a synergistic effect in attenuation of hyperlipidemia in HFD-fed mice, but *A. iwayomogi* extract (0.1% *w*/*w* diet) itself did not affect serum TG levels [12]. In addition to an appropriate dose necessary to reduce plasma TG levels, the differences in extract solvent and methods used in these two studies may also account for the reported variation in *A. iwayomogi* extract activity. Therefore, our study aimed to examine the protective effects of different doses of *A. iwayomogi* extract against hypertriglyceridemia induced by a HFD in mice and to uncover the molecular mechanisms involved in TG-rich lipoprotein metabolism in the liver.

## 2. Results

### 2.1. Chromatographic Analysis of A. iwayomogi Extract (AI)

**Figure 1:**
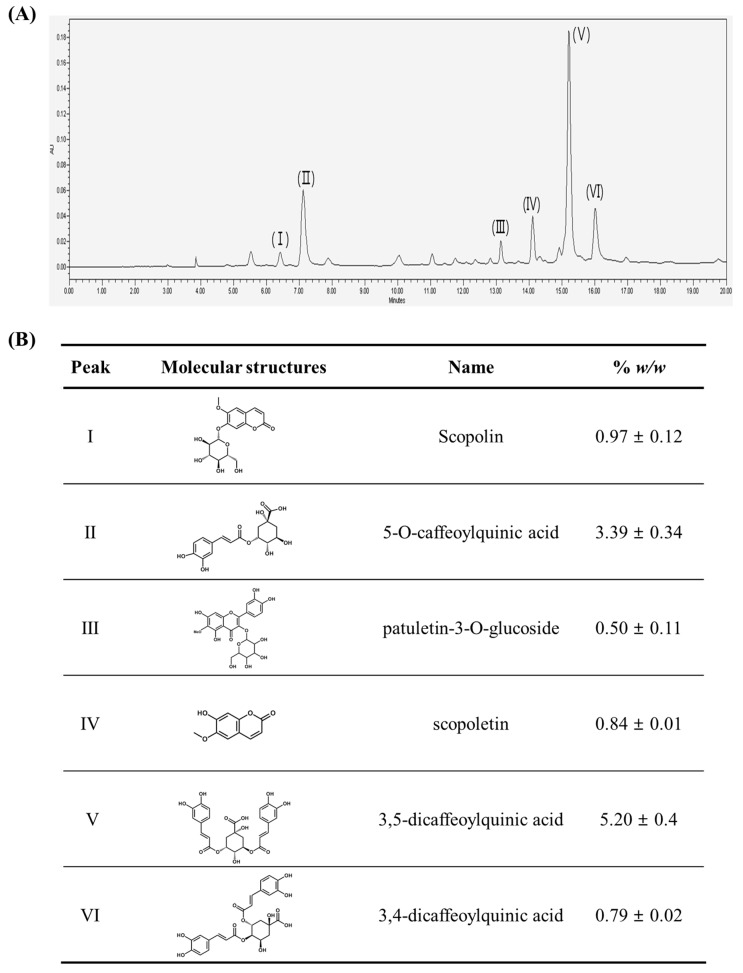
The contents of the individual bioactives identified in *Artemisia iwayomogi* extract (AI). (**A**) A representative high performance liquid chromatography (HPLC) chromatogram of the compound present in the extract is shown. The sample was run three times at 330 nm; (**B**) the molecular structures and concentrations (% *w*/*w*) of compounds identified in AI.

The high performance liquid chromatography (HPLC) chromatogram revealed that scopolin (I, 0.97 ± 0.12% *w*/*w*), 5-*O*-caffeoylquinic acid (II, 3.39 ± 0.34% *w*/*w*), patuletin-3-*O*-glucoside (III, 0.50 ± 0.11% *w*/*w*), scopoletin (IV, 0.84 ± 0.01% *w*/*w*), 3,5-dicaffeoylquinic acid (V, 5.20 ± 0.4% *w*/*w*) and 3,4-dicaffeoylquinic acid (VI, 0.79 ± 0.02% *w*/*w*) were the major organic components of *A. iwayomogi* extract (AI) (Figure 1).

### 2.2. AI Attenuates Hypertriglyceridemia in Mice Fed a High-Fat Diet

**Figure 2:**
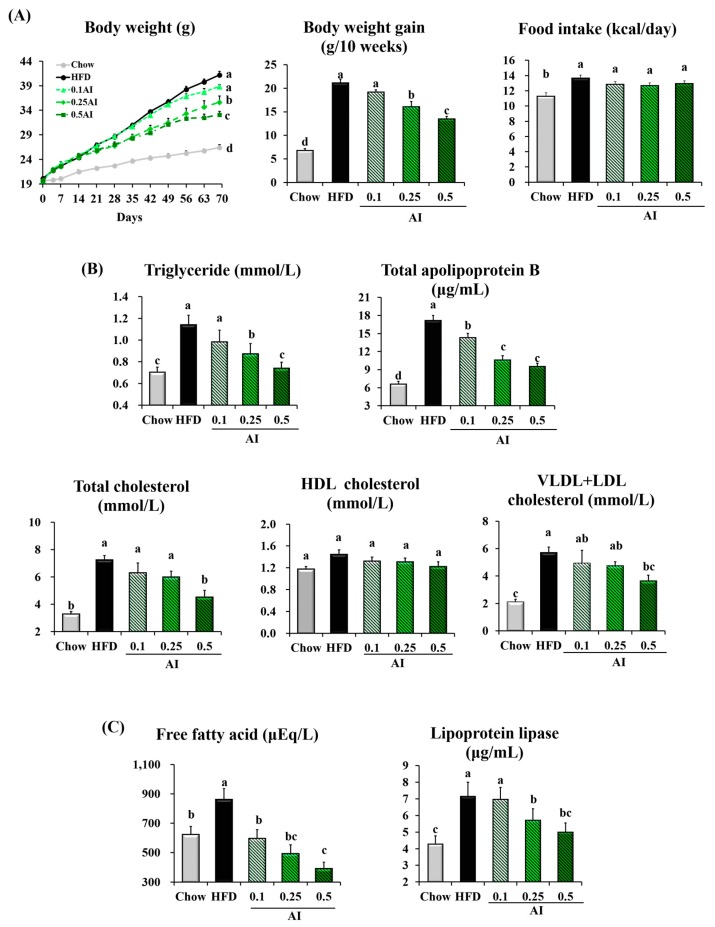
AI attenuates hypertriglyceridemia in high-fat diet (HFD)-fed mice. (**A**) Changes in body weight and food intake; (**B**) plasma levels of triglyceride, total apolipoprotein B, total cholesterol, very low density lipoprotein (VLDL) + LDL cholesterol, and high density lipoprotein (HDL) cholesterol; (**C**) plasma free fatty acid and lipoprotein lipase concentrations. Data are the mean ± standard error of the mean (SEM) of *n* = 8 mice. Statistical analysis was performed using one-way analysis of variance (ANOVA) and analyzed further by Duncan’s multiple range test. Different letters above the bars indicate significant differences among experimental groups (*p* < 0.05).

The metabolic actions of AI were evaluated following the administration of the extract by food admixture to C57BL/6N male mice challenged with a HFD. AI administration significantly reduced the body weight of HFD-fed mice in a dose- and time-dependent manners. After 10 weeks of treatment, 0.25% and 0.5% AI reduced the HFD-induced weight gain in mice by 24% and 36%, respectively. Importantly, this effect on body weight gain was not a result of altered feeding behavior (Figure 2A). HFD-induced hypertriglyceridemia was significantly improved in the mice that received 0.25% and 0.5% AI; the plasma levels of both TG and total apolipoprotein B (ApoB), the components of VLDL, were reduced. The decreased cholesterol concentration in the plasma of 0.5% AI-fed mice resulted mainly from a decrease in VLDL + low density lipoprotein (LDL) cholesterol. The plasma levels of free fatty acids and LPL, as determined by enzyme-linked immunosorbent assay (ELISA), were reduced by AI treatment in HFD-fed mice. The AI-induced improvements in lipid profiles appeared dose-dependent and were most significant at the highest dosage tested, 0.5% (Figure 2B,C).

### 2.3. AI Alleviates High-Fat Diet-Induced Hepatic Lipid Accumulation

**Figure 3:**
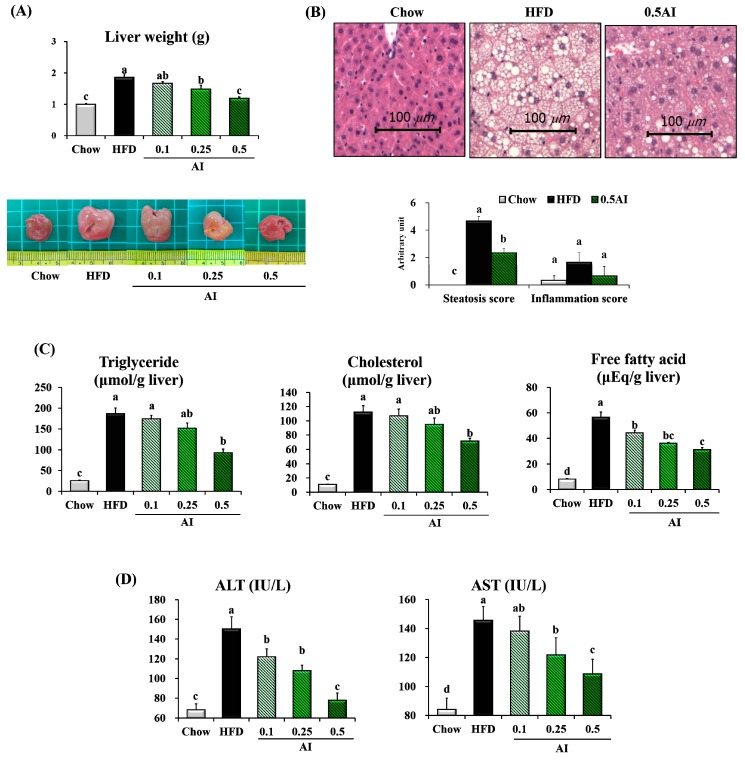
AI alleviates hepatic steatosis in HFD-fed mice. (**A**) Liver weights and gross morphology; (**B**) representative images of H&E-stained sections of liver (scale bar = 100 μm) and scores for hepatic steatosis and inflammation; (**C**) hepatic levels of triglyceride, cholesterol, and free fatty acid; (**D**) plasma aminotransferase (ALT) and aspartate aminotransferase (AST) activities; Data are the mean ± SEM of *n* = 8. Statistical analysis was performed using one-way ANOVA and analyzed further by Duncan’s multiple range test. Different letters above the bars indicate significant differences among experimental groups (*p* < 0.05).

The 10-weeks HFD significantly increased the size of the liver and lightened the color of the liver. AI supplementation clearly reversed the HFD-induced adverse changes in a dose-dependent manner. The mass of liver tissue from mice fed the HFD supplemented with 0.5% AI was 30% lower than those fed the HFD only (Figure 3A). In addition, the reduced liver mass observed in mice that received 0.5% AI was associated with a smaller number and size of lipid droplets, a feature that correlated with leanness and hepatic steatosis scores (Figure 3B). Although the hepatic inflammation scores followed a pattern similar to that of the steatosis scores, there were no statistically significant difference among the groups. The HFD-induced lipid accumulation in the liver was ameliorated by AI administration, as shown by the dose-dependent reduction of hepatic levels of TG, cholesterol, and FFAs (Figure 3C). Plasma alanine aminotransferase (ALT) and aspartate aminotransferase (AST) activities associated with hepatic injury were markedly increased in HFD-fed mice, but were reduced by AI administration in a dose-dependent manner (Figure 3D).

### 2.4. AI Activates Adiponectin-Adenosine Monophosphate-activated Protein Kinase (AMPK) Mediated Signaling Pathways in the Liver

**Figure 4:**
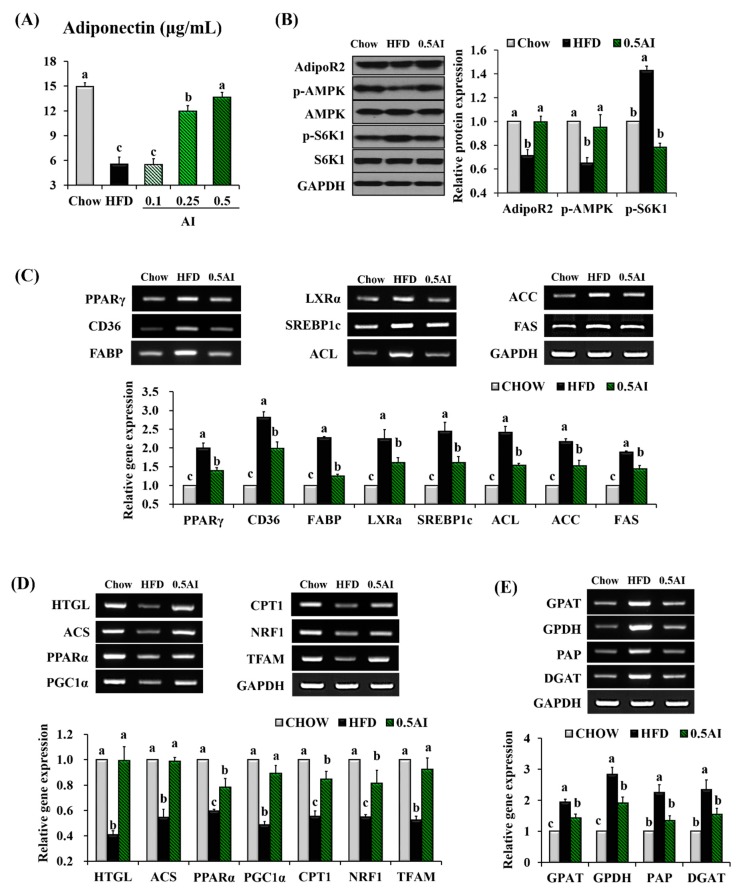
AI activates adiponectin-AMPK mediated signaling pathways in mice. (**A**) Plasma adiponectin concentrations; (**B**) representative western blot analysis and quantification of AdipoR2, total adenosine monophosphate-activated protein kinase (AMPK), p-AMPK, total S6K1, and p-S6K1 in the liver; Representative reverse transcription polymerase chain reaction (RT-PCR) product bands and quantitative comparison of mRNA expression for (**C**) lipogenesis, (**D**) lipolysis, fatty acid oxidation, inflammation and (**E**) triglyceride synthesis in the liver. Results are presented as the mean ± SEM. Statistical analysis was performed using one-way ANOVA and analyzed further by Duncan’s multiple range test. Different letters above the bars indicate significant differences among experimental groups (*p* < 0.05).

As plasma adiponectin level was completely elevated by AI administration (Figure 4A) and AMPK is one of the key activators of the fatty acid catabolism responsible for sensing low energy levels, we tested whether prolonged AI administration could modulate the adiponectin-AMPK pathways in the liver of HFD-fed mice. Immunoblotting and reverse transcription polymerase chain reaction (RT-PCR) analysis were conducted on the liver tissues of mice fed chow, HFD, and 0.5% AI-supplemented HFD. In HFD-mice, the observed changes (the downregulation of adiponectin receptor 2 ((AdipoR2) and phospho (p)-AMPK protein levels and the upregulation of p-S6 Kinase 1 (S6K1) protein expression) were completely normalized by the administration of AI (Figure 4B). The expression of lipogenic nuclear transcription factors, such as peroxisome proliferator-activated receptor γ (PPARγ), sterol regulatory element binding protein 1c (SREBP1c), and liver X receptor α (LXRα), was robustly downregulated in the liver of AI-treated mice compared with the HFD mice. The functional relevance of the AI-induced downregulation was confirmed by the decreased expression of direct target genes, including cluster of differentiation 36 (CD36), liver fatty acid binding protein 1 (FABP), ATP citrate lyase (ACL), acetyl-coenzyme A (CoA) carboxylases (ACC), and fatty acid synthase (FAS) (Figure 4C). Additionally, the supplementation of AI to the HFD had a strong positive impact on the expression of the regulators of hepatic lipolysis, such as hepatic triglyceride lipase (HTGL) and acyl CoA synthetase (ACS), and of the transcription factors involved in fatty acid oxidation and inflammation, such as peroxisome proliferator-activated receptor α (PPARα) and peroxisome proliferator-activated receptor-γ coactivator 1α (PGC1α), and their target genes including carnitine palmitoyltransferase 1 (CPT1), mitochondrial transcription factor A (TFAM), nuclear respiratory factor 1 (NRF1), tumor necrosis factorα (TNFα), and interlukin-6 (IL-6) (Figure 4D). The expression of TG synthesis enzymes, such as glycerol-3-phosphate acyltransferase (GPAT), glycerol-3-phosphate dehydrogenase (GPDH), phosphatidate phosphohydrolase (p), and diacylglycerol acyltransferase (DGAT), was significantly downregulated in the liver of AI-treated mice compared with the HFD-fed mice (Figure 4E).

### 2.5. AI Improves Insulin Resistance and Downregulates Molecules Involved in Very Low Density Lipoprotein Assembly

**Figure 5:**
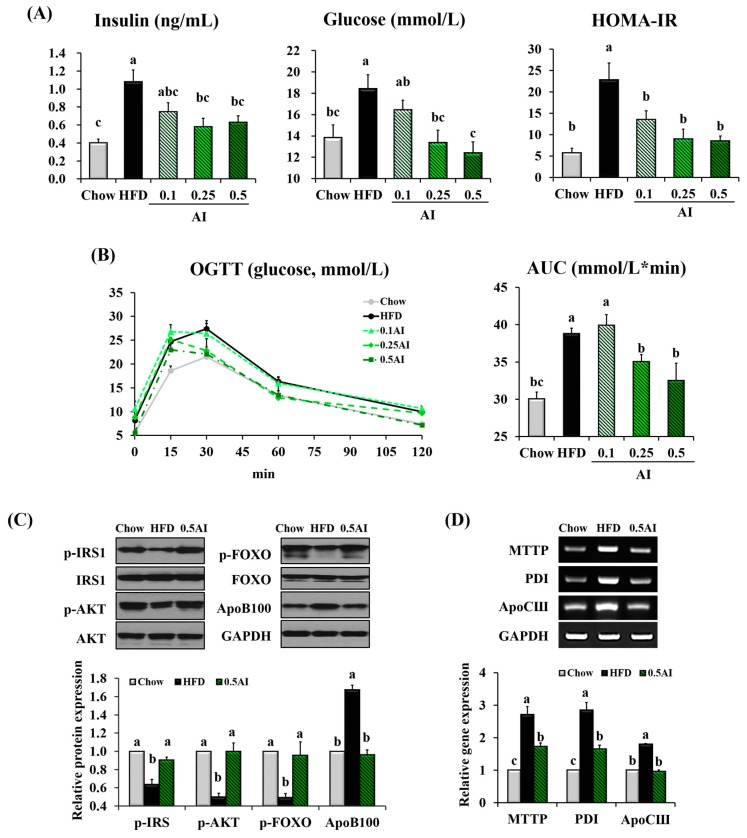
AI improves the hepatic insulin signaling pathway in mice. (**A**) Fasting plasma levels of insulin and glucose and homeostasis model assessment of basal insulin resistance (HOMA-IR)) index; (**B**) oral glucose tolerance test (OGTT) and area under the curve (AUC); (**C**) densitometric quantification of the phosphorylation of IRS1, AKT, FOXO, and ApoB100 in the liver; (**D**) hepatic expression of genes involved in VLDL assembly; Statistical analysis was performed using one-way ANOVA and analyzed further by Duncan’s multiple range test. Different letters above the bars indicate significant differences among experimental groups (*p* < 0.05).

AI also prevented the negative impact of the HFD on glucose homeostasis; fasting blood glucose and insulin levels were both reduced and the homeostasis model assessment of basal insulin resistance (HOMA-IR) value was decreased (Figure 5A). In addition, mice treated with AI showed improved tolerance to an oral glucose load, as demonstrated by a decreased area under the curve (AUC) value compared with the HFD-fed mice (Figure 5B). As the improved hepatic insulin sensitivity was associated with reduced VLDL assembly from TG and secretion by the liver, we evaluated whether AI administration could regulate the signaling molecules involved in insulin sensitivity and VLDL assembly in the liver. The downregulation of the phosphorylated form of insulin receptor substrate 1 (IRS1), protein kinase B (AKT), and forkhead box O1 (FOXO) proteins induced by HFD was completely reversed by AI administration. The protein expression of ApoB100 and gene expression of microsomal triglyceride transfer protein (MTTP), disulfide isomerase (PDI) and apolipoprotein CIII (ApoCIII), which were involved in VLDL packaging, were strongly downregulated in the livers of mice that received AI compared with those of HFD-fed mice (Figure 5C,D).

## 3. Discussion

In the present study, the ethanol extract of *A. iwayomogi* was supplemented to HFD-fed mice. At concentrations of 0.25% and 0.5% AI, dose-dependent improvements were observed in the major parameters of hypertriglyceridemia, such as plasma TG, FFA, ApoB, and LPL levels. The supplementation of 0.25% and 0.5% AI (equivalent to 250 and 500 mg·kg^−1^ body weight of mice/day, respectively) corresponded to intakes of approximately 1.2 g and 2.4 g/60 kg adult/day, respectively, when calculated on the basis of normalization to body surface area as recommended by Reagan-Shaw, Nihal, and Ahmad [14] and the US Food and Drug Administration (Available online: https://www.fda.gov/downloads/drugs/guidancecomplianceregulatoryinformation/guidances/ucm078932). The toxicological study of AI used in this experiment was conducted in male and female Sprague-Dawley rats after a 13-week repeated oral administration of 500, 1000, or 2000 mg·kg^−1^ body weight/day and revealed no treatment-related mortality or adverse effects, including clinical observations, motor activity assessment, body weight/weight gain, food consumption, ophthalmoscopy, clinical pathology, hematology, urinalysis, organ weights, gross pathology, or histopathology. Therefore, the no observed adverse effect level (NOAEL) of AI was estimated as equal to or greater than 2000 mg·kg^−1^ body weight/day in both male and female animals [15]. Similarly, Kim et al. [16] reported the NOAEL for fertility and early embryonic development of *A. iwayomogi* hot water extract as 2000 mg·kg^−1^ body weight/day for rats.

The HPLC chromatogram of AI revealed the presence of relatively high amounts of specific chlorogenic acids, such as 3,5-dicaffeoylquinic acid (5.2% *w*/*w*), 5-*O*-caffeoylquinic acid (3.4% *w*/*w*), and 3,4-dicaffeoylquinic acid (0.8% *w*/*w*). Chlorogenic acids are a family of esters formed between certain trans-cinnamic acids and (-)-quinic acid and are one of the most abundant polyphenols in the human diet; coffee, fruits, and vegetables are the major sources [17]. Approximately 71 different species of chlorogenic acid have now been identified from different sources [18,19]. Some in vitro and in vivo potential pharmacological properties have been reported, including hypoglycemic, antihypertensive, antiviral, antifungal, hepatoprotective, neuroprotective, and immunoprotective activities [20]. 5-*O*-Caffeoylquinic acid was reported to prevent mice from diet-induced obesity and obesity related metabolic syndromes, including hepatic steatosis, insulin resistance, and chronic inflammation [21,22,23]. Other types of chlorogenic acids, such as 3,5-dicaffeoylquinic acid and 3,4-dicaffeoylquinic acid, were also effective for the inhibition of oleic acid-induced lipid accumulation in human hepatoma cell lines (HepG2) [24,25]. Miyamae et al. [26] suggested that caffeoyl groups bound to quinic acid are important for activity and the more caffeoyl groups are bound to quinic acid, the higher accelerating activity on ATP production exhibits.

Besides chlorogenic acids, scopolin (0.97% *w*/*w*), scopoletin (0.84% *w*/*w*), and patuletin-3-*O*-glucoside (0.50% *w*/*w*) were also present in AI in considerable amount. Scopoletin and scopolin (a glucoside form of scopoletin) are coumarin compounds which have been identified in many different medicinal plants, including species of Scopolia, Brunfelsia, Solanum, and Mallotus, in addition to Artemisia. [27]. Data from our laboratory have shown that 0.02% (*w*/*w*) scopolin supplemented to HFD mice significantly prevented increments in body weight, and hepatic lipids levels [28]. Scopolin (100 mg·kg^−1^ body weight) ameliorated adjuvant-induced arthritis in rats by the downregulation of proinflammatory and proangiogenic cytokines, such as vascular endothelial growth factor, basic fibroblast growth factor-2, and interleukin-6 [29]. Scopoletin (0.05% *w*/*w*) effectively attenuated alcohol-induced hepatic steatosis in HFD-induced obese mice by the inhibition of lipogenesis through modulation of the AMPK–SREBP1c signaling pathway [30]. The involvement of scopoletin in the protection from the risk of Alzheimer’s disease has been suggested as this compound increased extracellular acetylcholine concentration, which is responsible for regulating learning and memory in rat brain. Patuletin-3-*O*-glucoside is commonly found in *A. iwayomogi*, but not in other species of Artemisia, such as *A. annua* and *A. capillaries* [31,32,33,34], and its physiological functions are still unknown. Further studies are needed to gain insight into the contributions of each phenolic compound to the AI-induced attenuation of hypertriglyceridemia in mice.

**Figure 6:**
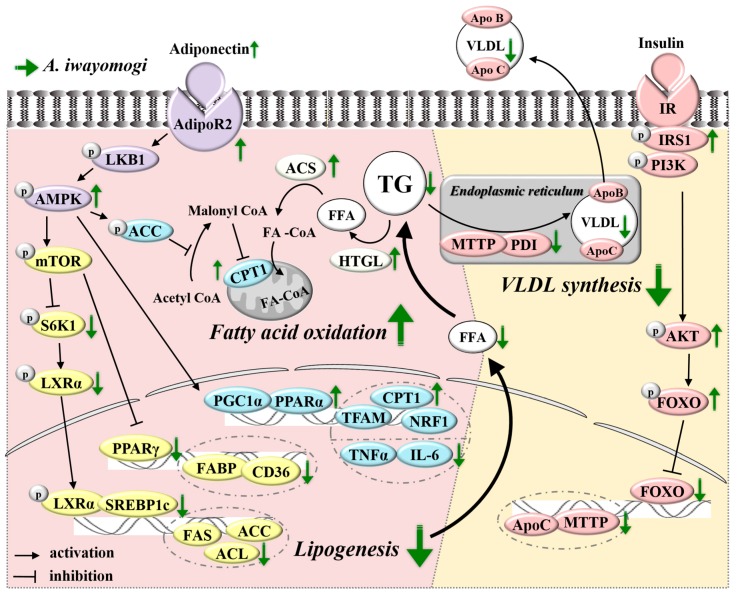
Schematic representation of mechanisms by which AI inhibits HFD-induced hypertriglyceridemia.

AMPK is a conserved intracellular energy sensor that has been implicated in the regulation of glucose and lipid homeostasis in hepatocytes [35]. HFD per se reduces circulating adiponectin level, which results in the decreased phosphorylation of AMPK via reduced liver kinase B1 (LKB1) phosphorylation in the liver. The inactivation of AMPK leads to increased phosphorylation and activation of mammalian target of rapamycin (mTOR)/S6K1, a subsequent increase in phosphorylation of LXRα [36], and the ultimate activation of SREBP1c, a lipogenic transcription factor. AMPK is also a well-established cellular energy sensor that switches on catabolic pathways, including fatty acid oxidation, and switches off anabolic pathways, including lipogenesis [37]. The HFD-induced inactivation of AMPK negatively regulated fatty acid oxidation through the inactivation of the transcription factors PGC1α and PPARα and the downregulation of their target genes, including CPT1, NRF1, and TFAM (Figure 6).

We observed that AI effectively reversed the HFD-induced decreases in plasma adiponectin level and hepatic AMPK phosphorylation in mice. The activation of AMPK following AI administration was accompanied by the inactivation of a downstream signaling cascade through S6K1, LXRα, SREBP1c, PPARγ, and their target genes involved in lipogenesis (FAS, ACL, ACC, FABP, and CD36), and by the activation of a signaling cascade through PGC1α, PPARα, and their target genes (CPT1, NRF1, and TFAM) involved in fatty acid oxidation (Figure 4 and Figure 6). The TG biosynthetic pathway in the liver is catalyzed by several enzymes: GPAT and DGAT catalyze the first and last steps in TG synthesis, respectively. GPDH is the enzyme responsible for the catalysis of the reversible redox conversion of dihydroxyacetone phosphate to glycerol 3-phosphate and phosphatidate phosphohydrolase (PAP) catalyzes the dephosphorylation of phosphatidate, which yields diacylglycerol and inorganic phosphate [38,39,40,41]. In the meantime, hepatic TGs are hydrolyzed by HTGL to form FFAs and the carboxyl group of the FFA forms a linkage with the thiol group of CoA to yield a fatty acyl-CoA; this process is catalyzed by ACS. The AI-induced attenuation of hypertriglyceridemia observed in HFD-fed mice was also associated with the downregulation of genes involved in TG biosynthesis (GPAT, GPDH, PAP, and DGAT) (Figure 4E) and the upregulation of genes related to TG hydrolysis (HTGL and ACS) in the liver (Figure 4D).

VLDL assembly in the liver is dependent on substrate availability and tightly regulated by insulin [42,43]. When hetero-dimerized with its small subunit PDI in the endoplasmic reticulum, MTTP catalyzed the transfer of TG to nascent ApoB, the rate-limiting step in hepatic VLDL production [44,45,46]. Under fasting conditions, hepatic VLDL production is induced, increasing VLDL secretion into the blood, whereas in response to postprandial insulin release, hepatic VLDL production is suppressed to limit plasma TG excursion [47,48,49,50]. The acute inhibition of VLDL production by insulin is critical for the rapid adaptation of the liver metabolism between fasting and refeeding states to maintaining plasma lipids within the physiological range. However, in an insulin-resistant state, hepatic VLDL production is elevated: the cell surface insulin receptor and intracellular mediators such as IRS1 and AKT are dephosphorylated, leading to decreased phosphorylation and degradation of FOXO and subsequent upregulation of MTTP and ApoC, which are key molecules in the assembly and intracellular trafficking of newly synthesized VLDL (Figure 6). We found that AI efficiently reversed the HFD-induced insulin resistance in mice as the fasting plasma levels of insulin and glucose and the AUC value from oral glucose loading were all significantly decreased. Furthermore, AI administration increased the phosphorylated form of proteins involved in insulin signaling, such as p-IRS1, p-AKT, and p-FOXO, and subsequently suppressed MTTP, PDI, and ApoCIII genes and ApoB100 protein in the liver (Figure 5 and Figure 6). The favorable effects of AI, comprising the improvement of HFD-induced insulin resistance and the downregulation of molecules involved in VLDL assembly in the liver, might be partially reflected in decreased plasma concentrations of triglyceride and ApoB protein (Figure 2B).

## 4. Materials and Methods

### 4.1. Preparation of AI and HPLC Analysis

The dried aerial part of *A. iwayomogi* was collected from Kyung-Dong Oriental Market in South Korea and identified by Wan Kyunn Whang. A voucher was deposited at the Botany Resources Laboratory at Chung-Ang University (Seoul, Korea). The *A. iwayomogi* was coarsely powdered and extracted with 50% ethanol on a rotary shaker at room temperature for 48 h. Liquid extract was separated and extracted with 50% ethanol at room temperature for additional 24 h. The ethanol extract was filtered with filter paper (Hyundai Micro Co., Seoul, Korea) and concentrated in a vacuum evaporator (N-11, Eyela, Tokyo, Japan). The mixture was sterilized for 10–20 min at 80–95 °C, dried in a spray dryer (B-290, Buchi, Germany) with maltodextrin.

For the HPLC of AI, the dried ethanol extract of *A. iwayomogi* (1 mg) was immersed in 1 mL 50% aqueous ethanol and filtered through a 0.2-μm membrane filter. HPLC analyses were performed using a Waters HPLC system (Waters Corporation, Milford, MA, USA) with an autosampler. Chromatographic separation was achieved using a Sunfire C18 column (4.6 mm × 250 mm, 5 μm inner diameter, Waters) at 30 °C with a flow rate of 0.9 mL·min^−1^ and a mobile phase composed of 0.1% (*v*/*v*) aqueous formic acid (A) and acetonitrile (B) using the following gradient elution: 85–84% A from 0–5 min; 84–72% A from 5–10 min; 72–69% A from 10–15 min, and 69–40% A from 15–20 min. The injection volume was 10 μL and the detection wavelength was set at 330 nm.

### 4.2. Animals and Diets

Five-week-old male C57BL/6N mice (18–20 g) were obtained from Orient Bio (Gyeonggi-do, Korea) and housed in a pathogen-free facility at Yonsei University (Seoul, Korea) at room temperature with a 12-h light/dark cycle. The study was approved by the Institutional Animal Care and Use Committee of the Yonsei Laboratory Animal Research Center (Permit no. IACUC-A-201402-142-01, 10 February 2014). The animals were allowed an acclimatization period of one week, during which they were fed a commercial diet (Purina Rodent Chow 5001, Nestlé Purina, St. Louis, MO, USA) with ad libitum access to tap water. Thereafter, the mice were divided into five groups (*n* = 8/group), which were each allocated to one of five experimental diets: chow diet (Chow), high-fat diet (HFD) (200 g fat·kg^−1^ body weight (170 g of lard and 30 g of corn oil) + 1% (*w*/*w*) cholesterol), and HFD supplemented with 0.1% AI, 0.25% AI, and 0.5% AI, respectively. The mice were subjected to the experimental diet for 10 weeks, during which time all animals were permitted ad libitum access to the diet and water. Throughout the experimental period, weekly body weight and daily food intake were measured. At the end of the experiment, mice were anaesthetized with 0.15 mL avertin (2.5% in tert-amyl alcohol) per 10 g body weight after a 12-h fast. Blood was drawn from the abdominal aorta into an ethylenediaminetetraacetic acid (EDTA)-coated tube. Plasma was subsequently obtained by centrifuging the blood at 2000× *g* for 15 min at 4 °C. The livers were excised, rinsed with phosphate-buffered saline (PBS), and weighed. A portion of each liver was fixed in 10% formalin for further analysis. The plasma and liver samples were stored at −80 °C until analysis.

The oral glucose tolerance test (OGTT) was performed 2 weeks prior to the end of the experimental period on 6 h-fasted mice. Glucose (2 g kg^−1^ bodyweight) was administered by oral gavage and the blood glucose levels were measured from the tail blood at 0, 15, 30, 60, 90, and 120 min after administration.

### 4.3. Biochemical Analysis

The plasma levels of TG, FFAs, total cholesterol, high density lipoprotein (HDL)-cholesterol, and glucose were enzymatically determined using commercial kits (Bio-Clinical System, Gyeonggi-do, Korea). Plasma LDL + VLDL cholesterol levels were calculated by subtracting the level of HDL cholesterol from the total cholesterol. Plasma total ApoB and LPL concentrations were measured using the commercially available mouse-specific enzyme-linked immunosorbent assay (ELISA) kits (MyBiosource, San Diego, CA, USA). The plasma concentrations of adiponectin and insulin were measured by using a commercially available mouse ELISA kit (Millipore, Billerica, MA, USA). The HOMA-IR, which was used to assess insulin resistance, was calculated as fasting plasma glucose × fasting plasma insulin/22.5. The plasma activities of AST and ALT were measured using commercial kits (Bio-Clinical System). After homogenization of the liver tissues, the lipids were extracted with chloroform: methanol (2:1) as previously described [51]. The chloroform phases were carefully removed, dried under nitrogen, and re-suspended in ethanol; TG, cholesterol and FFAs were assayed using same kits that were used for quantification in plasma.

### 4.4. Histological Examination

For hematoxylin and eosin (H&E) staining, formalin-fixed liver was embedded into paraffin and cut into 5 μm sections. An observer blinded to the treatment groups assigned steatosis (0–5 scale) and inflammation (0–3 scale) values to H&E-stained liver sections according to semi-quantitative pathological standards. Briefly, the pathological degree of steatosis was visually scored in five non-contiguous medium-power fields (MPFs, ×100) as follows: no steatosis = 0; minimal steatosis = 1; slight steatosis = 2; moderate steatosis = 3; marked steatosis = 4; severe steatosis = 5. In addition, the degree of lobular inflammation was scored as follows: no inflammation foci = 0; 1–2 inflammation foci per 100 × field = 1; 3–4 inflammation foci per 100 × field = 2; >4 inflammation foci per 100 × field = 3.

### 4.5. Semi-Quantitative RT-PCR Analysis

The total mRNA from liver samples was isolated using TRIzol reagent (Invitrogen, Carlsbad, CA, USA). RT-PCR was performed using a Superscript II kit (Invitrogen). The forward and reverse primer sets for target and internal marker genes are listed in Table S1 and the RT-PCR conditions were as follows: initial denaturation, 5 min, 94 °C; followed by 35–38 cycles of denaturation, 30 s, 94 °C; annealing, 30 s, 55 °C; and extension, 1 min, 72 °C; with a final extension period of 10 min at 72 °C. Next, 4 μL of each PCR reaction mixture was mixed with 1 μL 6× loading buffer and loaded onto a 2% agarose gel containing ethidium bromide. Glyceraldehyde-3-phosphate dehydrogenase (GAPDH) mRNA levels were used as an internal control.

### 4.6. Western Blot Analysis

The liver tissues were lysed in Western lysis buffer consisting of 100 mM Tris-HCl (pH 7.4), 5 mM EDTA, 50 mM NaCl, 50 mM sodium pyrophosphate, 50 mM NaF, 100 mM orthovanadate, 1% Triton X-100, 1 mM phenylmethylsulfonyl fluoride, 2 μg·mL^−1^ aprotinin, 1 μg·mL^−1^ pepstatin A, and 1 μg·mL^−1^ leupeptin. The samples were frequently vortexed during a 10-minute incubation period on ice and centrifuged for 20 min at 1300× *g*. The protein concentration of each supernatant was quantified using a protein assay reagent from the Bradford assay (Bio-Rad, Richmond, CA, USA) in accordance with the manufacturer’s instructions.

The proteins were loaded onto an 8% sodium dodecyl sulfate polyacrylamide gel electrophoresis (SDS-PAGE), and transferred to a nitrocellulose membrane (Amersham, Buckinghamshire, UK). After transfer, the membranes were blocked with 5% bovine serum albumin in Tris-buffered saline with 0.05% Tween 20 and probed with the specified primary antibodies (1:1000 dilution) overnight at 4 °C. Primary antibodies to the following proteins were tested: GAPDH, AMPK, p-AMPK (Thr172), S6K1, p-S6K1 (Thr389), IRS1, p-IRS1 (Ser302), AKT, p-AKT (Ser473), FOXO, p-FOXO (Ser253) (all obtained from Cell Signaling Technology, Danvers, MA, USA), AdipoR2, and ApoB100 (obtained from Abcam, Cambridge, UK). The membranes were washed and incubated with the appropriate secondary antibodies in Tris-buffered saline with 0.05% Tween 20 for 1 h. The blots were developed using an enhanced chemiluminescence detection kit (Amersham) in accordance with the manufacturer’s instructions.

### 4.7. Statistical Analysis

The results of body weight gain and plasma biochemistry are presented as the mean ± standard error of mean (SEM) of eight mice in each group. The RT-PCR and western blot data are shown as the mean ± SEM of three independent experiments (*n* = 2 or 3 per experiment) for each group, cumulatively including eight mice. Statistical analysis was performed using one-way ANOVA and analyzed further by Duncan’s multiple range test. Differences among experimental groups were considered to be statistically significant for values of *p* < 0.05.

## 5. Conclusions

Overall, our observations confirmed that AI improved hypertriglyceridemia, body weight gain, hepatic lipid accumulation, and insulin resistance during the administration of HFD in mice. The AI-induced improvement of hypertriglyceridemia appears to be exerted at two different levels: first, by decreasing lipid availability through stimulation of adiponectin-AMPK mediated signaling cascades; and second, by downregulating molecules involved in TG-rich lipoprotein assembly through improved hepatic insulin resistance. Thus, these findings supported the development of AI as a therapy or prevention for diet-induced hypertriglyceridemia.

## Supplementary Materials

Supplementary materials can be found at www.mdpi.com/1422-0067/18/8/1762/s1.

## Abbreviations

ACC	Acetyl-CoA carboxylases
ACL	ATP citrate lyase
ACS	Acyl coa synthetase
AdipoR2	Adiponectin receptor 2
AI	*Artemisia iwayomogi* extract
AKT	Protein kinase B
ALT	Alanine aminotransferase
AMP	Adenosine monophosphate
AMPK	Adenosine monophosphate-activated protein kinase
ANOVA	Analysis of variance
ApoB	Apolipoprotein B
ApoC	Apolipoprotein C
AST	Aspartate aminotransferase
AUC	Area under the curve
CD36	Cluster of differentiation 36
CoA	Coenzyme A
CPT1	Carnitine palmitoyltransferase 1
DGAT	Diacylglycerol acyltransferase
ELISA	Enzyme-linked immunosorbent assay
FABP	Liver fatty acid binding protein 1
FAS	Fatty acid synthase
FOXO	Forkhead box O1
GAPDH	Glyceraldehyde-3-phosphate dehydrogenase
GPAT	Glycerol-3-phosphate acyltransferase
GPDH	Glycerol-3-phosphate dehydrogenase
HDL	High density lipoprotein
HFD	High-fat diet
HPLC	High performance liquid chromatography
HOMA-IR	Homeostasis model assessment of basal insulin resistance
HTGL	Hepatic triglyceride lipase
IL-6	Interlukin-6
IRS1	Insulin receptor substrate 1
LDL	Low density lipoprotein
LKB1	Liver kinase B1
LPL	Lipoprotein lipase
LXRα	Liver X receptor α
MTTP	Microsomal triglyceride transfer protein
NOAEL	No observed adverse effect level
NRF1	Nuclear respiratory factor 1
p	phospho-
PAP	Phosphatidate phosphohydrolase
PDI	Protein disulfide isomerase
PGC1α	Peroxisome proliferator-activated receptor-γ coactivator 1α
PPARα	Peroxisome proliferator-activated receptor α
PPARγ	Peroxisome proliferator-activated receptor γ
RT-PCR	Reverse transcription polymerase chain reaction
S6K1	S6 kinase 1
SDS-PAGE	Sodium dodecyl sulfate polyacrylamide gel electrophoresis
SEM	Standard error of the mean
SREBP1c	Sterol regulatory element binding protein 1c
TFAM	Mitochondrial transcription factor A
TG	Triglyceride
TNFα	Tumor necrosis factor α
VLDL	Very low density lipoprotein
