# Supplementary material for: Artemisia Iwayomogi Extract Attenuates High-Fat Diet-Induced Hypertriglyceridemia in Mice: Potential Involvement of the Adiponectin-AMPK Pathway and Very Low Density Lipoprotein Assembly in the Liver
Source: Int J Mol Sci. 2017 Aug 12;18(8):1762. doi: 10.3390/ijms18081762 (PMC5578151; doi:10.3390/ijms18081762)
Supplement: Supplementary file 1 [file ijms-18-01762-s001.pdf]

Table S1. Primer sequences and RT-PCR conditions.

Gene Description	Primers	Sequences (5′→3′)	T_m (°C)	Size (bp)
Peroxisome proliferator-activated receptor γ (PPARγ)	F	TTCGGAATCAGCTCTGTGGA	55	142
	R	CCATTGGGTCAGCTCTTGTG		
Cluster of differentiation 36 (CD36)	F	TTAGTAGAACCGGGCCACGT	55	116
	R	CACAGTTCCGATCACAGCCC		
Liver Fatty acid binding protein 1 (FABP)	F	CCAGGAGAACTTTGAGCCATTC	55	71
	R	TGTCCTTCCCTTTCTGGATGA		
Liver X receptor (LXRα)	F	ATGAGACCAGCAAAGCCCTC	55	117
	R	AGTTAGAGGAGCCGAAGGGT		
Sterol regulatory element binding transcription factor 1 (SREBP1c)	F	TGTCACTAGCATGCCCCAAG	55	127
	R	CTGTCTCACCCCCAGCATAG		
ATP citrate lyase (ACL)	F	TCACATGACGGCCATTGTGG	60	192
	R	GCTGGCTTGGCTTTCTTTGC		
Acetyl-CoA carboxylase (ACC)	F	GCCATTGGTATTGGGGCTTAC	65	112
	R	CCCGACCAAGGACTTTGTTG		
Fatty acid synthase (FAS)	F	TCAGCAACACATCTCACGCC	55	154
	R	GTCTGCTCCTTGGGCTTGTC		
Hepatic triglyceride lipase (HTGL)	F	GCTGCCACTTCCTGGAACTC	55	192
	R	TGCAGCTCAGGCATAGACCT		
Acyl CoA synthetase (ACS)	F	ATCTGGTGGAACGAGGCAAG	55	158
	R	TCCTTTGGGGTTGCCTGTAG		
Peroxisome proliferator-activated receptor α (PPARα)	F	TGGGGATGAAGAGGGCTGAG	55	197
	R	ACGCAACGTAGAGTGCTGTG		
peroxisome-proliferator-activated receptor γ coactivator-1α (PGC1α)	F	AACCAGTACAACAATGAGCC	55	141
	R	TGTCAGTGCATCAAATGAGG		
Carnitine palmitoyl transferase 1 (CPT1)	F	CATGTATCGCCGCAAACTGG	55	114
	R	CCTGGGATGCGTGTAGTGTT		
Nuclear respiratory factor 1 (NRF1)	F	AAGCGATTGTACTCTGCATC	58	111
	R	CTTCCAGGATCATGCTCTTG		
Mitochondrial transcription factor A (TFAM)	F	TCGGAGCTCAGTCTGGGAAG	58	198
	R	AACACCTCCCCGATCCTCTG		
Glycerol-3-phosphate acyltransferase (GPAT)	F	GCACATTCTCTTCACCGCCA	60	163
	R	GCCTAGGTCGAAATCGCGAG		
Glycerol-3-phosphate dehydrogenase (GPDH)	F	CGCCTGGCCTTTCTCAATGT	60	197
	R	GGCAGCAGGCTGATTTCAGT		
Phosphatidate phosphohydrolase (PAP)	F	CGAGACCCAATCCCTGACCT	60	126
	R	GCAGGTTCCTGACTGTCAGC		
Diacylglycerol acyltransferase (DGAT)	F	GGCTGCTGCTACATGTGGTT	60	163
	R	CACCACAGGTTGACATCCCG		
Microsomal triglyceride transfer protein (MTTP)	F	ATCGGGTGGCTGTGGTGATA	55	161
	R	AGCCTTGTCCATCTGCATGC		
Protein disulfide isomerase (PDI)	F	GGTCGGCGAGTGCTCTG	55	178
	R	TCCTCCTCAGTGGCATTTTCT		
Apolipoprotein CIII (ApoCIII)	F	CTACTCCAGGTAATGCCCCTG	58	118

	R	CGTACCATGAGTCCCAAGCC		
Tumor necrosis factor α (TNFα)	F	TGTCTCAGCCTCTTCTCATT	55	156
	R	AGATGATCTGAGTGTGAGGG		
Interleukin 6 (IL-6)	F	CCCCAATTTCCAATGCTCTCC	58	141
	R	CGCACTAGGTTTGCCGAGTA		
Glyceraldehyde-3-phosphate dehydrogenase (GAPDH)	F	AGAGAGGCCCTATCCCAACTC	55	193
	R	CCTCCCCATACACACCCTCC		
